# Single‐nucleus RNA sequencing reveals RUNX1 regulation of muscle hypertrophy through PI3K/AKT/mTOR pathway

**DOI:** 10.1002/imt2.70093

**Published:** 2025-11-20

**Authors:** Chenxu Wang, Junjie Ma, Yibin Wang, Rui Liu, Chenxi Zhang, Qingyuan Li, Lixin Zhang, Qihang Hou, Xiaojun Yang

**Affiliations:** ^1^ College of Life Sciences Northwest A&F University Yangling Shaanxi China; ^2^ College of Animal Science and Technology Northwest A&F University Yangling Shaanxi China

## Abstract

We used snRNA‐seq to construct a high‐resolution atlas of pectoral muscle development in broiler chickens from neonatal to adult stages. This analysis revealed pronounced molecular heterogeneity among satellite cells across developmental phases and uncovered a previously uncharacterized *Runx1* ^+^ satellite cell subpopulation. By integrating pseudotime trajectory reconstruction, gene set enrichment analysis, dynamic expression profiling and loss‐of‐function assays, we established a critical regulatory role for RUNX1 in muscle hypertrophy. Mechanistically, RUNX1 promotes myotube hypertrophy by transcriptionally repressing *Pik3r1*, thereby reducing PI3K p85α levels, destabilizing PTEN, and activating the PI3K/AKT/mTOR signaling cascade, which enhances protein synthesis and drives myotube growth.

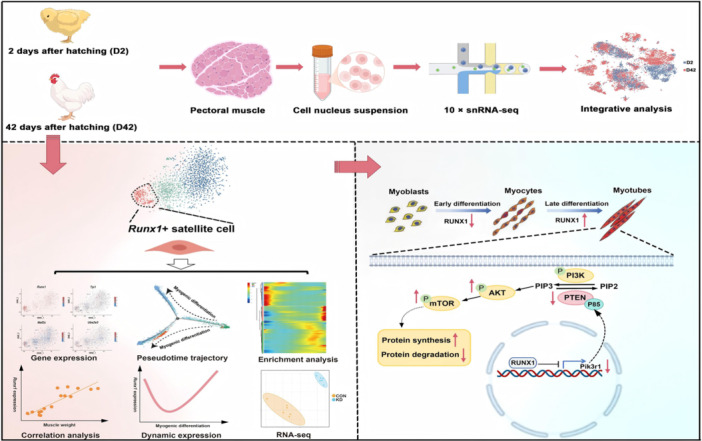

To the editor,

Skeletal muscle is fundamental for maintaining key physiological functions in vertebrates, including movement, metabolism, and respiration [1, 2]. In livestock and poultry, the development of skeletal muscle directly determines meat quality and yield, making it a central focus of animal science research [3, 4, 5]. Skeletal muscle is a heterogeneous tissue composed of diverse cellular populations [6]. Among these, myogenic cells represent the core compartment responsible for muscle growth and regeneration. This intrinsic heterogeneity forms the foundation for the tissue's developmental and functional complexity [7]. Although myogenic cell diversity and its regulatory dynamics during myogenesis have been extensively characterized in model organisms, these advances have not yet been fully translated to livestock and poultry species [8, 9].

Runt‐related transcription factor 1 (*Runx1*), a member of the RUNX family, has been shown to coordinately regulate myogenic gene expression with MyoD and c‐Jun during muscle injury [10, 11, 12]. Furthermore, *Runx1* expression is enriched in myonuclei during acute skeletal muscle hypertrophy [13]. However, the molecular mechanisms underlying its role in hypertrophy, particularly downstream signaling pathways and regulatory interactions, remain largely unexplored.

In this study, we performed single‐nucleus RNA sequencing (snRNA‐seq) of pectoral muscle from Arbor Acres white‐feather broiler to generate stage‐specific single‐nucleus transcriptomic profiles of pectoral muscle development. This analysis revealed distinct satellite cell subpopulations and identified a previously uncharacterized *Runx1* ^+^ satellite cell subset. Functional experiments further demonstrated that *Runx1* promotes myotube hypertrophy by activating the PI3K/AKT/mTOR signaling pathway. Together, our findings identify *Runx1* as a critical regulator of muscle growth and hypertrophy, providing potential molecular targets for combating muscle atrophy and offering genetic strategies to improve meat production in poultry.

## MAJOR CELL POPULATIONS OF MUSCLE IN BROILERS DURING POSTNATAL MYOGENESIS

**Figure 1:**
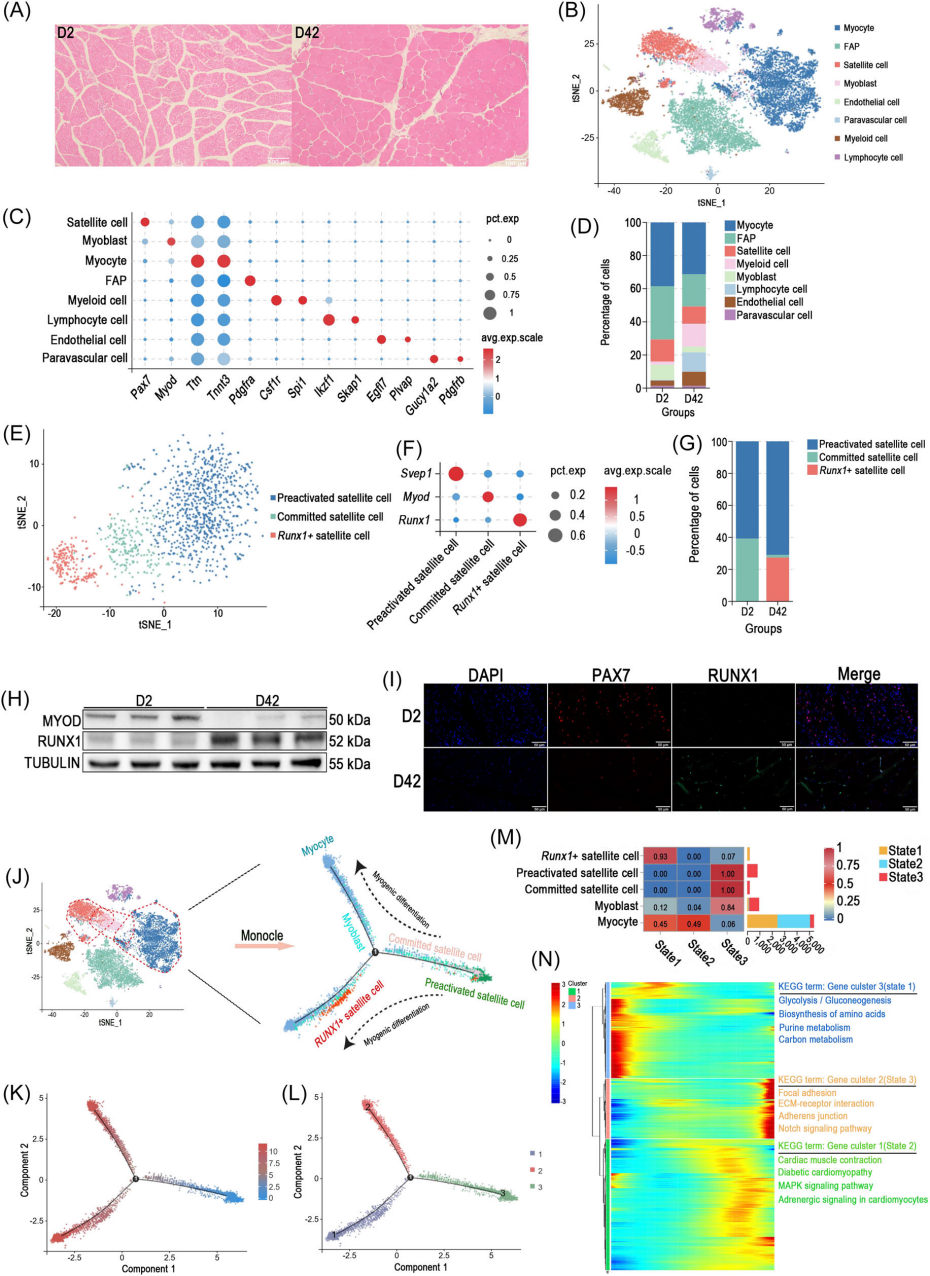
Construction and analysis of single‐nucleus transcriptome atlas of pectoral muscle tissue of postnatal broilers. (A) Representative H&E‐stained section of pectoral muscle from Arbor Acres broilers at D2 (Day 2 post‐hatch) and D42 (Day 42 post‐hatch); Scale bars, 100 μm. (B) t‐SNE (t‐distributed stochastic neighbor embedding) visualization of major cell types by transcriptional similarity. Cells were annotated using canonical marker genes; each dot represents one cell. (C) Bubble plots showing the mean expression of canonical marker genes across cell types. (D) Bar plot showing the proportion of each cell types per group. (E) t‐SNE plots showing the distribution of satellite cell subpopulations. (F) Bubble plots of mean expression of marker genes for satellite cell subpopulations. (G) Bar plot showing the proportions of satellite cell subpopulations per group. (H) Immunoblots for MYOD and RUNX1 in pectoral muscle of broilers at D2 and D42. (I) Representative immunofluorescence images of muscle sections stained for *Runx1* ^+^ satellite cell markers at D2 and D42. RUNX1 (green), PAX7 (red), and DAPI (blue). Scale bars, 50 μm. (J) Cells from satellite cell, myoblast, and myocyte clusters were subsisted (left) and subjected to pseudotime analysis to infer myogenic‐lineage trajectories (right). (K) Myogenic differentiation trajectory colored by pseudotime value. (L) Myogenic differentiation trajectory colored by differentiation states. (M) Quantitative distribution of myogenic cells across different differentiation states. (N) Heatmap of differentially expressed genes (DEGs) across the three differentiation states (left); DEGs were grouped into three clusters with characteristic expression patterns. Kyoto Encyclopedia of Genes and Genomes (KEGG) pathway analysis for each cluster is shown (right) with representative enriched biological processes.

To comprehensively characterize postnatal myogenesis dynamics in broiler chickens, we collected pectoral muscle samples from male Arbor Acres broilers at Day 2 (D2) and Day 42 (D42) post‐hatch. Morphological analysis revealed pronounced muscle hypertrophy from D2 to D42, as evidenced by enlarged cross‐sectional area [14] (Figure 1A, Figure S1A). To investigate cellular heterogeneity and transcriptional dynamics, we performed snRNA‐seq on pectoral muscle at both time points, generating a high‐quality data set comprising 15,455 nuclei and 48,737 genes (Table S4).

Principal component analysis followed by t‐distributed stochastic neighbor embedding (t‐SNE) dimensionality reduction revealed nineteen distinct cell clusters (Figure S1B,C). Based on established cell‐specific marker genes, we annotated eight major populations: satellite cells, myoblasts, myocytes, fibro‐adipogenic progenitors (FAPs), endothelial cells, paravascular cells, myeloid cells, and lymphocytes (Figure 1B, Figure S1D,E). The accuracy of these annotations was supported by bubble plots showing cluster‐specific expression of canonical marker genes (Figure 1C). Kyoto Encyclopedia of Genes and Genomes (KEGG) pathway enrichment analysis of upregulated genes in each cluster further confirmed distinct biological functions (Figure S1G, Table S5).

Quantitative analysis of cellular composition revealed marked shifts during postnatal muscle development. The proportion of myogenic cells progressively declined with age, as reflected by reduced frequencies of satellite cells and myoblasts, suggesting attenuated myogenic activity. In contrast, immune cells, including myeloid cells and lymphocytes, exhibited substantial expansion (Figure 1D, Figure S1F), suggesting maturation of the muscle's immune responsiveness during injury [15].

## UNBIASED DISSECTION OF MYOGENIC SUBPOPULATIONS

We re‐clustered myogenic cells to resolve higher‐resolution heterogeneity. This analysis identified two established satellite cell subpopulations, preactivated and committed satellite cells, and uncovered a previously uncharacterized *Runx1* ^+^ satellite cell subset (Figure 1E). Marker genes defining each subpopulation are summarized in Figure 1F. Consistent with the continuum of myogenic differentiation [16, 17], preactivated and committed satellite cells together constituted the entire satellite cell population at D2. From D2 to D42, the frequency of committed satellite cell decreased while that of preactivated cells increased, reflecting a developmental shift from active proliferation toward quiescence during muscle maturation (Figure 1G,H, Figure S2A,B).

Remarkably, despite the global suppression of myogenesis at D42, we identified a previously uncharacterized *Runx1* ^+^ satellite cell population. Immunofluorescence staining of muscle sections of D42 confirmed nuclear co‐localization of RUNX1 and PAX7 (Figure 1I). Although *Runx1* ^+^ satellite cells expressed the myogenic regulators *Mef2c* and *Mef2d*, their transcriptional program was distinct from that of committed satellite cells. *Runx1* ^+^ satellite cells exhibited elevated expression of glycolytic enzymes and endoplasmic reticulum (ER)‐associated functional genes (Figure S2C, Table S6), indicating robust protein biosynthesis and quality control activity. KEGG pathway enrichment further highlighted a unique metabolic signature characterized by enrichment in ER protein processing, glycolysis/gluconeogenesis, ubiquitin‐mediated proteolysis, and amino acid biosynthesis (Figure S2D).

We also re‐clustered myocytes (Figure S3A) and distinguished fast and slow muscle‐cell populations based on canonical markers (Figure S3B,C). Quantitative analysis revealed that slow muscle cells predominated at D2, whereas fast muscle cells became the dominant population by D42, indicating a developmental shift in myofiber composition (Figure S3D). In terms of metabolic preference, fast muscle cells were biased toward glycolysis, while slow muscle cells favored oxidative phosphorylation (Figure S3E,F, Table S7).

Together, these results reveal that *Runx1⁺* satellite cells represent a distinct subpopulation with specialized metabolic features that may contribute to maintaining satellite cell function during muscle growth.

## PSEUDOTIME TRAJECTORY OF THE MYOGENIC LINEAGE

To resolve the differentiation hierarchy among myogenic cells during postnatal muscle development, we constructed a pseudotime trajectory encompassing preactivated satellite cells, committed satellite cells, *Runx1* ^+^ satellite cells, myoblasts, and myocytes (Figure 1J). Ordering cells by pseudotime revealed three distinct differentiation states (Figure 1K,L, Table S8). Preactivated satellite cells, which exhibited the highest stemness potential, occupied the earliest position along the trajectory, displaying the lowest pseudotime values. Committed satellite cells progressed along the same continuum and populates differentiation state 3, continuous with preactivated satellite cells. In contrast, *Runx1* ^+^ satellite cells diverged onto a distinct branch that exclusively occupied differentiation state 1 (Figure 1J,M).

To elucidate the molecular basis of these lineage bifurcations, we performed KEGG pathway analysis on genes upregulated within each differentiation state, with emphasis on state 1. We found that genes enriched in state 1 were associated with glycolysis/gluconeogenesis, biosynthesis of amino acids, purine metabolism, and carbon metabolism (Figure 1N). Genes exhibiting dynamic expression patterns along the trajectory are shown in Figure S4 and Table S9. As a newly defined subpopulation, *Runx1* ^+^ satellite cells followed a unique differentiation route and exhibited a distinct transcriptional and metabolic profile. Compared with other myogenic cells, they displayed specialized metabolic features, particularly enhanced carbon and protein metabolism.

## EXPRESSION PATTERN OF *RUNX1* IN MUSCLE TISSUE AND MYOBLASTS

**Figure 2:**
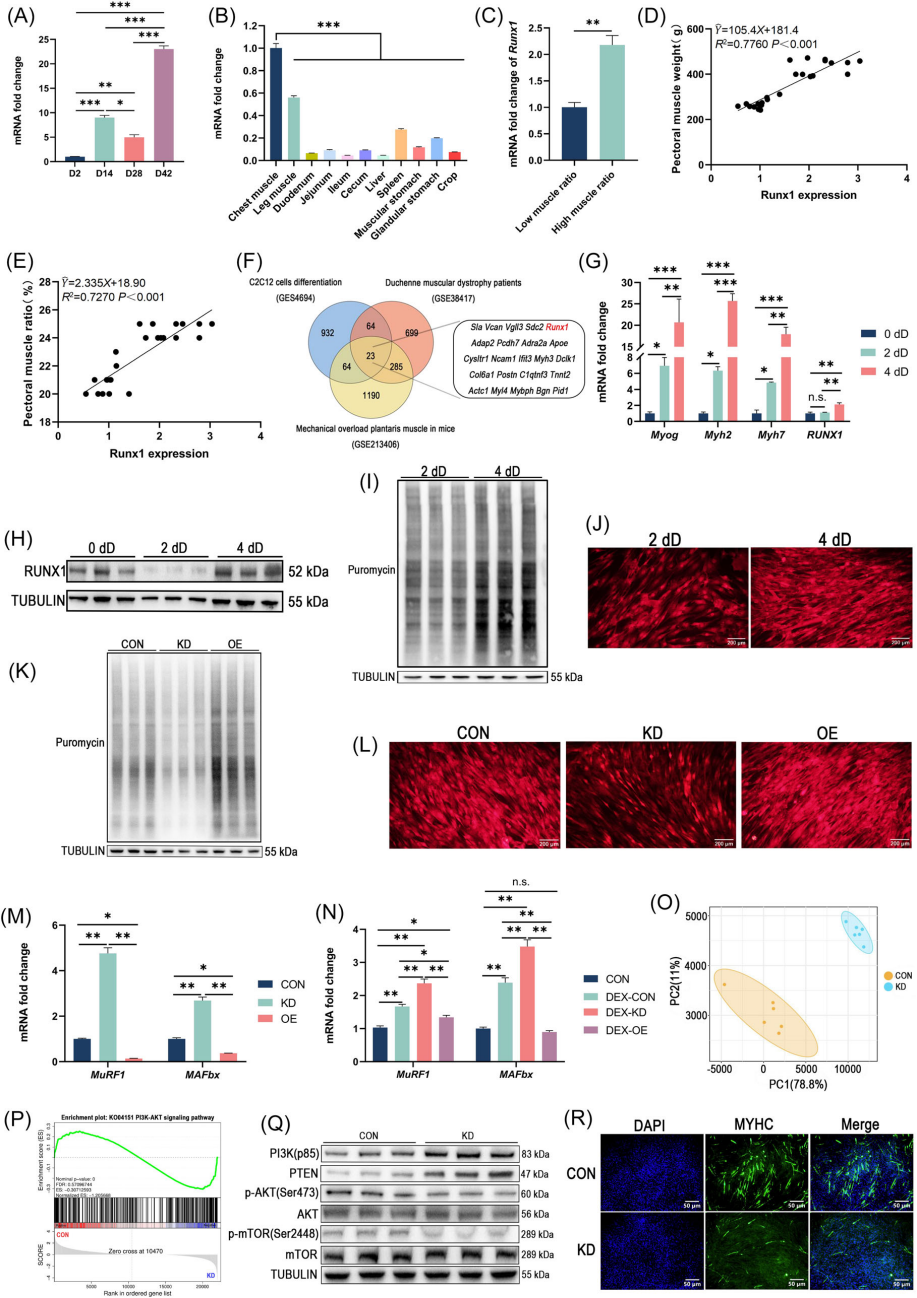
RUNX1 promotes muscle hypertrophy through activation of the PI3K/AKT/mTOR signaling pathway. (A) mRNA expression of *Runx1* in the pectoral muscle of broilers at Day 2, Day 14, Day 28, and Day 42 post‐hatch. (B) mRNA expression of *Runx1* in major organs of broilers. (C) mRNA expression of *Runx1* in pectoral muscle of high and low pectoral muscle ratios. Simple linear regression analyses showing correlation between *Runx1* mRNA expression and (D) pectoral muscle weight and (E) pectoral‐muscle ratio. (F) Venn diagram showing 23 overlapping DEGs among three independent myogenesis‐related microarray datasets. (G) mRNA expression of *Myog*, *Myh2*, *Myh7*, and *Runx1* in myoblasts at 0 dD (proliferation phase), 2 dD (2 days of differentiation phase), and 4 dD (4 days of differentiation phase). (H) Immunoblot of total RUNX1 protein in myoblasts at 0 dD, 2 dD, and 4 dD. (I) Immunoblot analysis of puromycin‐labled proteins in myoblasts at 2 dD and 4 dD. (J) Representative l‐homopropargylglycine fluorescence images of myoblasts at 2 dD and 4 dD. Scale bars, 200 μm. (K) Immunoblot of puromycin‐labeled proteins in control (CON), *Runx1* knockdown (KD), and overexpression (OE) myoblasts at 4 dD. (L) Representative l‐homopropargylglycine fluorescence images of CON or *Runx1* KD and *Runx1* OE myoblasts at 4 dD. Scale bars, 200 μm. (M) mRNA expression of *MuRF1* and *MAFbx* in CON or *Runx1* KD and *Runx1* OE myoblasts at 4 dD. (N) mRNA expression of *MuRF1* and *MAFbx* in CON or *Runx1* KD and *Runx1* OE myoblasts at 4 dD treated with vehicle (DMSO) or dexamethasone. (O) Principal component analysis of CON or *Runx1* KD myoblasts at 4 dD. (P) Significantly enriched PI3K/AKT/mTOR signaling pathway identified by GSEA (Gene Set Enrichment Analysis). (Q) Immunoblot of indicated proteins in CON and *Runx1* KD myoblasts at 4 dD. (R) Representative immunofluorescence images of MYHC (green) of CON and *Runx1* KD myoblasts at 4 dD; nuclei counterstained with DAPI (blue). Scale bars, 50 μm. Values are presented as mean ± SEM (*n* = 3, 6, or 24). Statistical significance was determined using two‐tailed Student's *t*‐test (two groups) or one‐way ANOVA followed by Duncan's multiple range post hoc test (multiple groups). Asterisks denote significant differences (*p < 0.05, **p < 0.01, and ***p < 0.001).

To investigate the role of *Runx1* in muscle physiology, we examined its expression across developmental stages and multiple tissues in broiler chickens. *Runx1* expression exhibited an oscillatory increase from D2 to D42, peaking at D42 (Figure 2A). Among the tissues analyzed, *Runx1* expression was specifically enriched in muscle, particularly in the pectoral muscle (Figure 2B). To further evaluate the association between *Runx1* and muscle growth, individuals with high and low pectoral muscle ratios were selected for analysis. Broilers with a high pectoral muscle ratio exhibited significantly higher *Runx1* expression (Figure 2C), and *Runx1* transcript levels positively correlated with both pectoral muscle weight and ratio (Figure 2D,E). These results indicate a strong association between *Runx1* expression and pectoral muscle development in broilers.

To extend this observation, we integrated three independent microarray datasets related to myogenesis from the Gene Expression Omnibus (GEO). *Runx1* was consistently identified among the overlapping genes shared across all datasets (Figure 2F, Table S10), suggesting its involvement in myogenic differentiation during muscle regeneration and hypertrophy.

To verify this hypothesis in vitro, myoblasts were induced to differentiate for 2 days (2 dD) and 4 days (4 dD). Expression of differentiation‐ and myotube‐related genes was significantly elevated at 4 dD compared to 2 dD, indicating enhanced myotube formation at the later stage of differentiation (Figure 2G). We next assessed *Runx1* expression during proliferation (0 dD) and differentiation (2 dD and 4 dD). *Runx1* mRNA levels showed no significant change between 0 dD and 2 dD but increased significantly at 4 dD (Figure 2G). At the protein level, RUNX1 expression decreased at 2 dD upon differentiation induction and subsequently rebounded at 4 dD (Figure 2H).

## RUNX1 PROMOTES MYOTUBE PROTEIN SYNTHESIS AND SUPPRESSES PROTEASOMAL DEGRADATION

Given the differential expression of *Runx1* in proliferating (0 dD) versus differentiating (2 dD) myoblasts and the discordance between its mRNA and protein levels, we hypothesized that RUNX1 underwent proteasomal degradation during proliferation‐to‐differentiation transition. Proteasome inhibition with bortezomib confirmed proteasome‐dependent loss of RUNX1 (Figure S5A,B). We next generated *Runx1* knockdown (KD) and overexpression (OE) myoblasts by lentiviral transduction. EdU proliferation assays (Figure S5C,D), together with measurements of CCND1 (Figure S5E,F) and MYOG (Figure S5G,H) expression, demonstrated that RUNX1 promoted myoblast proliferation and restrained differentiation relative to controls (CON). Notably, *Runx1* protein levels rebounded at 4 dD in parallel with elevated *Runx1* mRNA expression, coincident with robust myotube formation. We therefore posited that restored *Runx1* expression late in differentiation supports myotube hypertrophy.

To test this, we quantified protein synthesis using puromycin and l‐homopropargylglycine incorporation [18]. Protein synthesis in myotubes was significantly higher at 4 dD than at 2 dD (Figure 2I,J, Figure S6A,B). Relative to CON, *Runx1* knockdown impaired protein synthesis in myotubes, while *Runx1* overexpression enhanced it (Figure 2K,L, Figure S6C,D). At the transcriptional level, *Runx1* knockdown upregulated the muscle‐specific E3 ubiquitin ligases *MAFbx* and *MuRF1*, whereas *Runx1* overexpression suppressed their expression (Figure 2M). Finally, in a dexamethasone‐induced in vitro atrophy model, dexamethasone increased *MAFbx* and *MuRF1* expression in myoblasts as expected [19]; *Runx1* knockdown exacerbated this induction, whereas *Runx1* overexpression attenuated it (Figure 2N).

## RUNX1 PROMOTES MYOTUBE HYPERTROPHY VIA PI3K/AKT/MTOR SIGNALING

To define the mechanism by which RUNX1 drives myotube hypertrophy, we performed RNA‐seq on control and *Runx1* knockdown myotubes at 4 dD. *Runx1* depletion significantly altered the transcriptome (Figure 2O), yielding 1600 differentially expressed genes (|log_2_FC|≥1 and *p* < 0.05), including 1021 upregulated and 579 downregulated genes (Table S11). Gene Set Enrichment Analysis revealed significant downregulation of pathways related to muscle cytoskeleton, calcium signaling, ribosome biogenesis, PI3K‐AKT signaling, glycolysis/gluconeogenesis, and positive regulation of skeletal muscle tissue development in knockdown cells (Figure 2P and Figure S6E).

Given the central role of the PI3K/AKT/mTOR pathway in protein synthesis and the elevated *Pik3r1* expression observed in KD cells [20] (Figure S6F), we queried the JASPAR database (https://jaspar.genereg.net) for RUNX1‐binding motifs within the *Pik3r1* promoter and identified putative binding sites (Figure S6G). This suggests that *Runx1* may regulate PI3K/AKT/mTOR signaling by modulating *Pik3r1* transcription. Consistent with this hypothesis, immunoblotting showed increased PI3K p85α and PTEN protein levels, accompanied by decreased p‐AKT and p‐mTOR in KD cells (Figure 2Q and Figure S6H). Furthermore, MYHC immunofluorescence revealed reduced MYHC abundance (Figure 2R) and a lower myotube fusion index in KD versus control cells (Figure S6I), consistent with impaired hypertrophy capacity.

Together, these findings demonstrate that RUNX1 is specifically expressed in satellite cells of adult broilers and activates the PI3K/AKT/mTOR signaling pathway to promote muscle hypertrophy. However, the precise molecular mechanisms underlying RUNX1‐*Pik3r1* promoter interactions require further investigation, and future studies should employ additional animal models to validate the physiological relevance of this regulatory axis in muscle biology.

In summary, this study established a snRNA‐seq atlas of postnatal broiler pectoral muscle development and identified a previously uncharacterized *Runx1* ^+^ satellite cell subpopulation. Functional analyses revealed that RUNX1 plays a pivotal role in promoting muscle development and hypertrophy through PI3K/AKT/mTOR signaling activation. These findings establish RUNX1 as a crucial regulator of muscle growth and a potential molecular target for enhancing muscle mass and combating muscle atrophy.

## AUTHOR CONTRIBUTIONS

**Chenxu Wang:** Conceptualization; methodology; data curation; visualization; writing—original draft; formal analysis; validation; investigation; writing—review & editing. **Junjie Ma:** Software; formal analysis; writing—review & editing. **Yibin Wang:** visualization; conceptualization. **Rui Liu:** Resources; investigation. **Chenxi Zhang:** Software; data curation. **Qingyuan Li:** Investigation; formal analysis. **Lixin Zhang:** Supervision; project administration; resources. **Qihang Hou:** Supervision; methodology; project administration; writing—review & editing. **Xiaojun Yang:** Supervision; funding acquisition; project administration; resources; writing—review & editing; conceptualization. All authors have read the final manuscript and approved it for publication.

## CONFLICT OF INTEREST STATEMENT

The authors declare no conflicts of interest.

## ETHICS STATEMENT

The ethics application (NWAFAC1008) was approved by the Animal Care and Use Committee of Northwest A & F University.

## Supporting information


**Figure S1.** Single‐nucleus transcriptional profiling of pectoral muscle cells in broilers at D2 and D42.
**Figure S2.** Characterization of the satellite cell subpopulations.
**Figure S3.** Characterization of the myocyte subpopulations.
**Figure S4.** The dynamic expression of representative genes from differentiation states.
**Figure S5.** *Runx1* promotes myoblasts proliferation and inhibits differentiation.
**Figure S6.** *Runx1* activates the PI3K/AKT/mTOR signaling pathway through transcriptional regulation of *Pik3r1*.

**Table S1.** Composition and nutrient levels of basal diets, as‐fed basis.
**Table S2.** Antibodies used for this study.
**Table S3.** RT‐qPCR primer specifications.
**Table S4.** Basic quality control of sequencing data.
**Table S5.** Upregulated genes in maior cell type.
**Table S6.** Upregulated genes in satellite cell subpopulation.
**Table S7.** Upregulated genes in myocyte subpopulation.
**Table S8.** Pseudotime information.
**Table S9.** DEGs of differentiation states.
**Table S10.** DEGs of 3 microarray datasets.
**Table S11.** DEGs of CON and KD cells.

## Data Availability

The data that support the findings of this study are openly available in GSA at https://ngdc.cncb.ac.cn/bioproject/browse/PRJCA044373, reference number PRJCA044373. All the sequencing data have been deposited in GSA under submission number CRA028890 for snRNA‐seq raw data (https://ngdc.cncb.ac.cn/gsa/browse/CRA028890). The data and scripts used are saved in GitHub (https://github.com/1wangchenxu/scRNAseq). Supplementary materials (methods, figures, tables, graphical abstract, slides, videos, Chinese translated version, and updated materials) may be found in the online DOI or iMeta Science http://www.imeta.science/.
