# Supplementary material for: Single‐nucleus RNA sequencing reveals RUNX1 regulation of muscle hypertrophy through PI3K/AKT/mTOR pathway
Source: Imeta. 2025 Nov 20;4(6):e70093. doi: 10.1002/imt2.70093 (PMC12747532; doi:10.1002/imt2.70093)
Supplement: Supplementary file 1 — Figure S1. Single‐nucleus transcriptional profiling of pectoral muscle cells in broilers at D2 and D42. Figure S2. Characterization of the satellite cell subpopulations. Figure S3. Characterization of the myocyte subpopulations. Figure S4. The dynamic expression of representative genes from differentiation states. Figure S5. Runx1 promotes myoblasts proliferation and inhibits differentiation. Figure S6. Runx1 activates the PI3K/AKT/mTOR signaling pathway through transcriptional regulation of Pik3r1. [file IMT2-4-e70093-s002.docx]

# Supporting information to

# Single-nucleus RNA sequencing reveals RUNX1 regulation of muscle hypertrophy through PI3K/AKT/mTOR pathway

Chenxu Wang^1^, Junjie Ma^2^, Yibin Wang^2^, Rui Liu^2^, Chenxi Zhang^2^, Qingyuan Li^2^, Lixin Zhang^1,*^, Qihang Hou^2,*^, Xiaojun Yang^2,*^

^1^College of Life Sciences, Northwest A&F University, Yangling, Shaanxi 712100, China

^2^College of Animal Science and Technology, Northwest A&F University, Yangling, Shaanxi 712100, China

* Corresponding author: E-mail: [zhanglixin@nwsuaf.edu.cn (Lixin Zhang);](mailto:zhanglixin@nwsuaf.edu.cn;) E-mail:qihanghou1992@163.com (Qihang Hou); E-mail: [yangxj@nwsuaf.edu.cn](mailto:yangxj@nwsuaf.edu.cn) (Xiaojun Yang)

## METHODS

### Animals

A total of 12 1-day-old healthy male Arbor Acres broilers with no significant difference in body weight were provided by Dacheng company (Xi 'an, China). All broilers were kept in environmentally controlled poultry house with double-floor cages. Broilers were allowed access to fresh water and feeds freely. The composition and nutritional levels of the broiler basal diet was shown in the Table S1. Pectoral muscle tissues were collected on day 2 and day 42 post-hatching, immediately snap-frozen in liquid nitrogen, and stored at -80°C for long-term preservation.

### Pectoral muscle nuclei isolation

To isolate nuclei, the collected tissue was transferred to a Dounce homogenator and were homogenized in precooled lysis buffer (0.25 M sucrose, 5 mM CaCl, 3 mM MgAc, 10 mM Tris-HCl pH 8.0, 1 mM DTT, 0.1 mM EDTA, 1 × Protease Inhibitor (Thermo Scientific, Catalog number 78425), 1 U/μL RiboLock RNase Inhibitor (Thermo Scientific, Catalog number 00381). The tissue homogenate was washed with nuclei washing buffer (PBS containing 0.04 % BSA, 0.2 U/μL RiboLock RNase Inhibitor, 500 mM mannitol, and 0.1 mM PMSF Protease Inhibitor (Thermo Scientific, Catalogue 36978), followed by filtration through a 70 um cell sieve. The nuclei fraction was mixed with an equal volume of 50 % iodixanol solution (0.16 M sucrose, 10 mM NaCl, 3 mM MgCl, 10 mM Tris-HCl pH 7.4, 1 U/μL RiboLock, RNase Inhibitor, 1 mM DTT, 0.1 mM PMSF Protease Inhibitor) to a final concentration of 25 %, then 1mL of a 33 % iodixanol solution was added to the bottom of the tube, followed by a 30 % iodixanol solution to the top. This solution was mixed by flipping it 10 times and then centrifuged at 500 × g for 8 min at 4 ℃. After removing the myelin layer from the top of the gradient, nuclei were collected from the 30 % iodixanol interface. The nuclei were resuspended and filtered through a 40 um filter to remove cell debris nuclei clumps. The total number and concentration of nuclei and the proportion of intact nuclei with nuclear membrane were calculated. Finally, the nuclei concentration was adjusted to 700-1200 nuclei/μL.

### Construction and sequencing of the snRNA-seq library

Nuclei suspensions were loaded onto a 10 × Genomics GemCode single cell instrument and processed according to the manufacturer's protocol to produce single-cell Gel Bead-In-EMulsions (GEMs). Sequencing libraries were constructed using the Chromium Next GEM single-cell 3' Reagent Kit v3.1 (10 × Genomics), which contains sequencing primers, a 10 × barcode, a unique molecular identifier (UMI), and polydT primers. Cell lysis, RNA extraction, cDNA synthesis, and amplification were performed automatically on an Illumina sequencing platform (Genedenovo Biotechnology, Guangzhou, China).

### Data processing and analysis of snRNA-seq

### Data processing

Raw BCL files were converted to FASTQ files, and individual samples were then aligned and quantified using 10 × Genomics Cell Ranger software (version 3.1.0). Briefly, reads with low-quality barcodes and UMIs were filtered out and then mapped to the reference genome. First, at least 50 % of reads mapping to the transcriptome and intersecting an exon were considered. Second, nucleus with unusually high numbers of UMIs ( ≥ 5000) or with less than 200 or more than 8000 genes detected were filtered out. After the QC filters, a total of 51,159 nucleus from independent experiments were analyzed by Seurat software [1].

### Dimensionality reduction and visualization

The nucleus by gene matrices for each sample were individually imported to Seurat (version 3.1.1) for downstream analysis [2]. To minimize the effects of batch effect and behavioral conditions on clustering, we used Harmony to aggregate all samples. Principal component analysis (PCA) was used to reduce the dimension of the combined data [3]. After computing the shared nearest neighbor graph, the cells were clustered using the graph clustering method Louvain based on PCA dimensionality reduction data. For visualization of clusters, t-distributed Stochastic Neighbor Embedding (t-SNE) were generated using the same PCs.

### Differentially expressed gene analysis and gene functional enrichment

Expression value of each gene in given cluster were compared against the rest of cells using Wilcoxon rank sum test. Significant up-regulated genes were identified using a number of criteria. First, genes had to be at least 1.28-fold overexpressed in the target cluster. Second, genes had to be expressed in more than 25 % of the cells belonging to the target cluster. Third, *P* value is less than 0.05. The identity of the cell clusters was assigned by manual annotation using marker genes identified from the literature. DEGs were analysed by Gene Ontology (GO) and Kyoto Encyclopedia of Genes and Genomes (KEGG) enrichment analyses. Hypergeometric test was used to detect the enrichment significance of differentially expressed genes (DEGs) in metabolic and signal transduction pathways. Gene Ontology (GO) and Kyoto Encyclopedia of Genes and Genomes (KEGG) enrichment analyses were used to identify which DEGs were significantly enriched in GO terms or metabolic pathways. GO terms and KEGG pathway analyses using the hypergeometric test were performed to identify significantly enriched metabolic pathways or signal transduction pathways enriched in DEGs. GO terms and KEGG pathways with false discovery rates *p* < 0.05 were considered significantly different. Pathways satisfying *p* < 0.05 were considered to be significantly enriched in DEGs.
**Construction of single cell trajectories**

The gene expression matrix generated by 10 × Genomics was imported into monocle (version 2.6.4) to construct cell differentiation trajectories, and the cell trajectories were visualized for different differentiation states, different samples and different cell subsets. state refers to a segment in the tree structure generated by monocle [4].

### mRNA sequencing

Total RNA was extracted from *Runx1* knockdown and control C2C12 cells at 4 days of differentiation using TRIzol reagent. The cDNA library construction, sequencing, and transcriptome data analysis were performed by Gene Denovo Biotechnology Co., Ltd. (Guangzhou, China). The DESeq2 method was used to identify differentially expressed genes using fold change ≥ 2.0 and *p* < 0.05 as the criteria. GO (http://geneontology.org) and KEGG (http://www.genome.jp/kegg) of differentially expressed genes function and signaling pathways cluster analysis.

### Hematoxylin and eosin staining and immunofluorescence multiple staining

Muscle tissues were collected and fixed in 4 % paraformaldehyde for 24 h. After gradient dehydration and paraffin embedding, muscle tissue was cut into 3-micron thick sections. Hematoxylin and eosin (H&E) staining was performed using hematoxylin and eosin staining. A representative visual field was selected to observe the morphological changes of muscle fibers during muscle development. For immunofluorescence, Sections and cells were permeabilized with Triton X-100 for 15 min after fixation, blocked in 5 % bovine serum albumin in PBS for 30 min. The cells were then incubated with primary antibodies overnight at 4 ℃. After incubation with the secondary antibody for two hours, the sections and cells were blocked with antifading sealant containing 4’,6-diamidino-2-phenylindole (P0131, Beyotime, China). Images were acquired with a Zeiss AX10 microscope (Zeiss, Germany) and analysed by Image-Pro Plus 6.0 (Media Cybernetics, USA). The primary antibodies used for immunofluorescence were listed in Table S2.

### Quantitative Real-Time PCR analysis

Total RNA of muscle tissues was extracted with TRIzol reagent (Hunan Accurate Biology Engineer Co.,Ltd, China ) according to the manufacturer’s instructions. Spectrophotometer was used to determine RNA concentration and quality. Then total RNA was transcribed into cDNA using the Primescript RT master mix kit (Takara Bio Inc., Dalian, China). RT-qPCR was conducted in an iCycler iQ5 multicolor real-time PCR detection system (Bio-Rad Laboratories) using SYBR Green PCR Master Mix (Takara, Dalian, China). Procedure was as followed: 95 °C for 30 s, followed by 40 amplification cycles of 95 °C for 15 s, 60 °C for 30 s. Relative quantification of the target gene expression was quantified using the Livak method and normalized to the expression of control group [5]. The primers used for RT-qPCR in this study were listed in Table S3.

### Cell culture

C2C12 cell line purchased from the China Cell Bank Resource Center and primary myoblasts were cultured in high glucose Dulbecco's Modified Eagle medium (11965092, Gibco, USA) supplemented with 10 % fetal bovine serum (FBS) (10099141c, Gibco, USA). After the myoblasts were cultured to 80 %-90 % confluence, the growth medium was replaced with differentiation medium consisting of high-glucose Dulbecco's modified Eagle medium supplemented with 2 % horse serum (26050070, Gibco, USA).

### Construction and packing of lentivirus

For stable gene expression in C2C12 myoblasts, the *Runx1* gene was cloned into LV5 EF-1a/GFP&Puro vector. Recombinant lentiviruses were produced by cotransfecting HEK293T cells with the lentiviral expression and packaging plasmids (pGag/Pol, pRev and pVSV-G) respectively. Medium containing virus was harvested at 72 hours after cell transfection, and the virus was filtered through a 0.45 μm filter before infection.

### Cell proliferation assay

Cells were seeded at a density of 10^5^ in 12-well plates, and when cells reached 50 % to 60 % confluence, cells were incubated with EdU and cell proliferation was measured using the BeyoClick™ EDU-488 Cell Proliferation Assay Kit (C0071S, beyotime, China) following the manufacturer's instructions.

### Protein synthesis detection assay

In brief, L-homopropargylglycine is a methionine analogue that can be incorporated into newly synthesized proteins instead of methionine during protein synthesis [6]. L-homopropargylglycine, on the other hand, can perform click reactions. The newly synthesized proteins are labeled with the corresponding fluorescent probes by the click reaction, so that the newly synthesized proteins can be detected by the appropriate fluorescence detection equipment. Cells were seeded in 12-well plates and differentiated four days later using differentiation medium. Cells were incubated with Methionine free and serum free medium containing L-homopropargylglycine for 0.5 hours and the protein synthesis was measured using the BeyoClick™ L-homopropargylglycine-555 Protein Synthesis Assay Kit (P1206S, beyotime, China) following the manufacturer's instructions.

### Western blotting assay

Cell and tissue protein was extracted using radioimmunoprecipitation assay (RIPA) buffer containing phenylmethylsulfonyl fluoride (PMSF) protease inhibitors. Western blot assays were performed as previously reported. In brief, Equal amounts of protein (40-80 ug) were separated by 12 % SDS-polyacrylamide gel electrophoresis and transferred onto PVDF membrane. Membranes were blocked with 5 % (w/v) skimmed milk powder or bovine serum albumin for 1 h at room temperature, followed by incubation with primary antibodies at 4 °C overnight. After washing with TBST (3 × 10 min), membranes were incubated with HRP-conjugated secondary antibodies for 1 h at room temperature. Protein bands were visualized using enhanced chemiluminescence (ECL) substrate and imaged with a ChemiDoc Imaging System (Bio-Rad). Band intensities were quantified using ImageJ software, normalized to GAPDH, and expressed as fold changes relative to the control group. The information of primary and secondary antibodies was listed in Table S2.

### Statistical analysis

Data were expressed as mean ± standard error of mean, two-tailed *t* test was used for comparison between the two groups and one-way analysis of variance (ANOVA) with Duncan’s post hoc test was used for multiple comparisons. Statistical significance was defined as **p* < 0.05, ***p* < 0.01 and ****p* < 0.001. All data were analyzed using SPSS Statistics 20 (SPSS, Chicago, IL, USA).

## REFERENCES

1. Christopher S. McGinnis, Lyndsay M. Murrow, Zev J. Gartner. 2019. “DoubletFinder: Doublet Detection in Single-Cell RNA Sequencing Data Using Artificial Nearest Neighbors.” *Cell Systems* 8: 329-+. <https://doi.org/10.1016/j.cels.2019.03.003>

2. Andrew Butler, Paul Hoffman, Peter Smibert, Efthymia Papalexi, Rahul Satija. 2018. “Integrating single-cell transcriptomic data across different conditions, technologies, and species.” *Nature Biotechnology* 36: 411-+. <https://doi.org/10.1038/nbt.4096>

3. Tim Stuart, Andrew Butler, Paul Hoffman, Christoph Hafemeister, Efthymia Papalexi, William M. Mauck, Yuhan Hao, Marlon Stoeckius, Peter Smibert, Rahul Satija. 2019. “Comprehensive Integration of Single-Cell Data.” *Cell* 177: 1888-+. <https://doi.org/10.1016/j.cell.2019.05.031>

4. Cole Trapnell, Davide Cacchiarelli, Jonna Grimsby, Prapti Pokharel, Shuqiang Li, Michael Morse, Niall J. Lennon, Kenneth J. Livak, Tarjei S. Mikkelsen, John L. Rinn. 2014. “The dynamics and regulators of cell fate decisions are revealed by pseudotemporal ordering of single cells.” *Nature Biotechnology* 32: 381-U251. <https://doi.org/10.1038/nbt.2859>

5. Sean C. Taylor, Katia Nadeau, Meysam Abbasi, Claude Lachance, Marie Nguyen, Joshua Fenrich. 2019. “The Ultimate qPCR Experiment: Producing Publication Quality, Reproducible Data the First Time.” *Trends in Biotechnology* 37: 761-774. <https://doi.org/10.1016/j.tibtech.2018.12.002>

6. Spencer J. Spratt, Kenichi Oguchi, Keisuke Miura, Masato Asanuma, Hina Kosakamoto, Fumiaki Obata, Yasuyuki Ozeki. 2022. “Probing Methionine Uptake in Live Cells by Deuterium Labeling and Stimulated Raman Scattering.” *Journal of Physical Chemistry B* 126: 1633-1639. <https://doi.org/10.1021/acs.jpcb.1c08343>


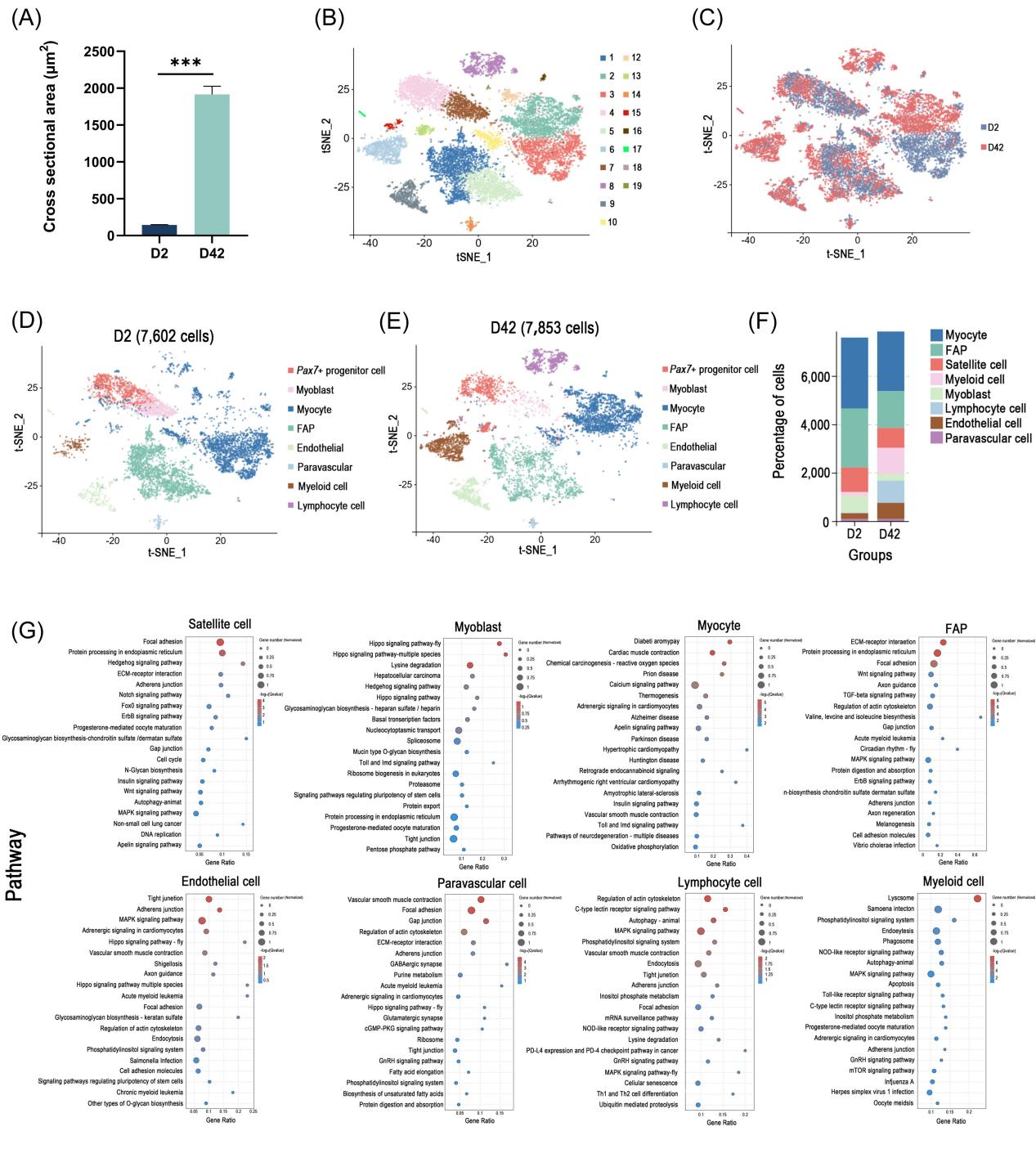


Figure S1. Single-nucleus transcriptional profiling of pectoral muscle cells in broilers at D2 and D42. (A) Quantification of cross sectional area of the pectoral muscle from Arbor Acres broilers on D2 (day 2 post-hatch) and D42 (day 42 post-hatch). (B) t-SNE plot visualization of all 15,455 cells categorized by cell clusters and (C) samples. Each dot is a cell. (D) t-SNE plot visualization of major cell types of D2 and (E) D42. (F) Bar plot showing the quantity of different cell types within each sample. (G) KEGG analysis of the up-regulated differentially expressed genes in cell types. Values are expressed as mean ± SEM (n = 6). The two-tailed Student's *t*-test were used for two groups statistical analysis; asterisks denote significant differences (**p* < 0.05, ***p* < 0.01 and ****p* < 0.001).


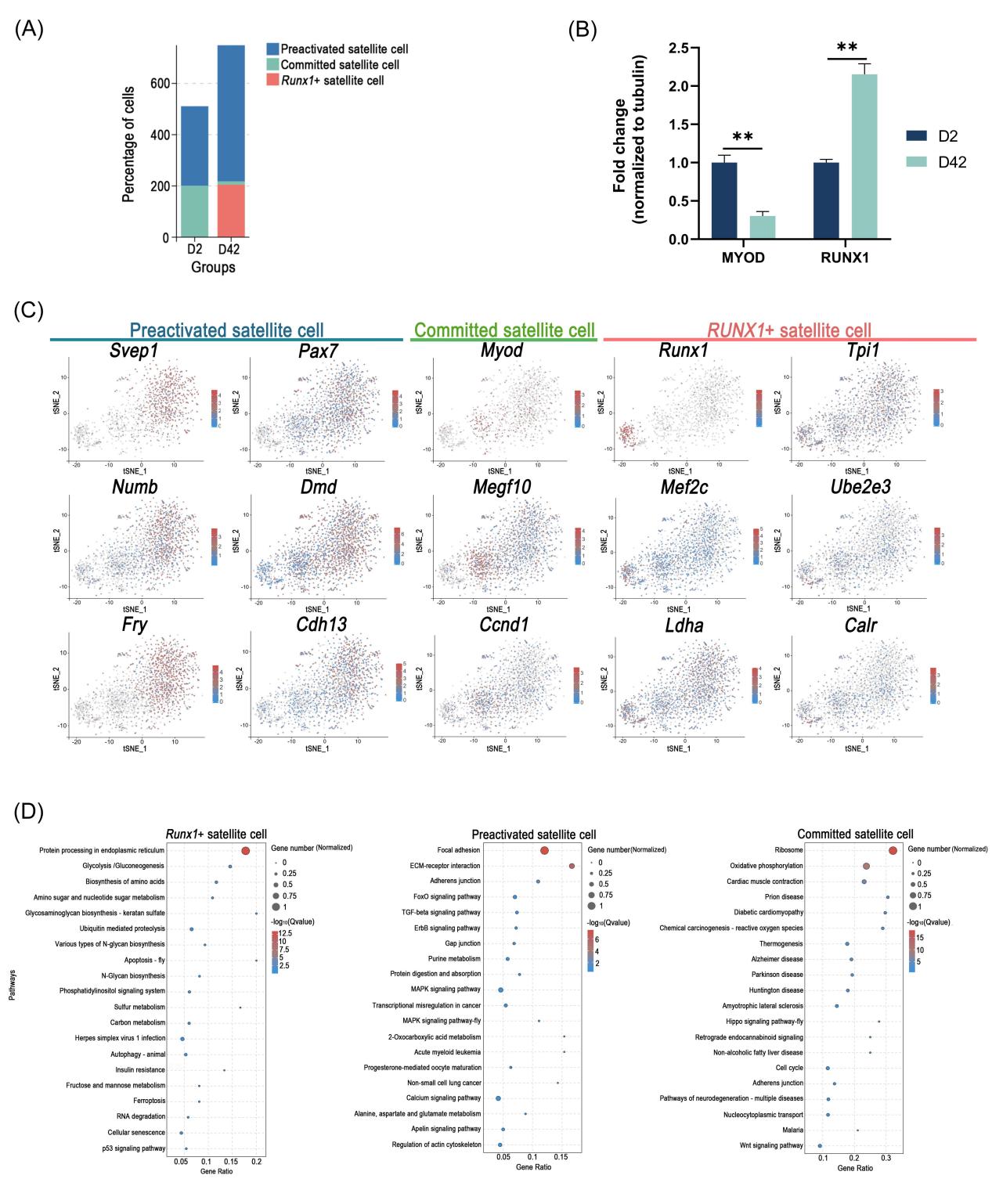


Figure S2. Characterization of the satellite cell subpopulations. (A) Bar plot showing the quantity of different satellite cell subpopulations within each sample. (B) Quantification of total protein of MYOD and RUNX1 in pectoral muscle of broilers on D2 and D42. (C) t-SNE plot showing the expression levels of representative genes in preactivated satellite cell, committed satellite cell and *Runx1*^+^ satellite cell. (D) KEGG analysis of the up-regulated differentially expressed genes in satellite cell subpopulations. Values are expressed as mean ± SEM (n = 3). The two-tailed Student's *t*-test were used for two groups statistical analysis; asterisks denote significant differences (**p* < 0.05, ***p* < 0.01 and ****p* < 0.001).


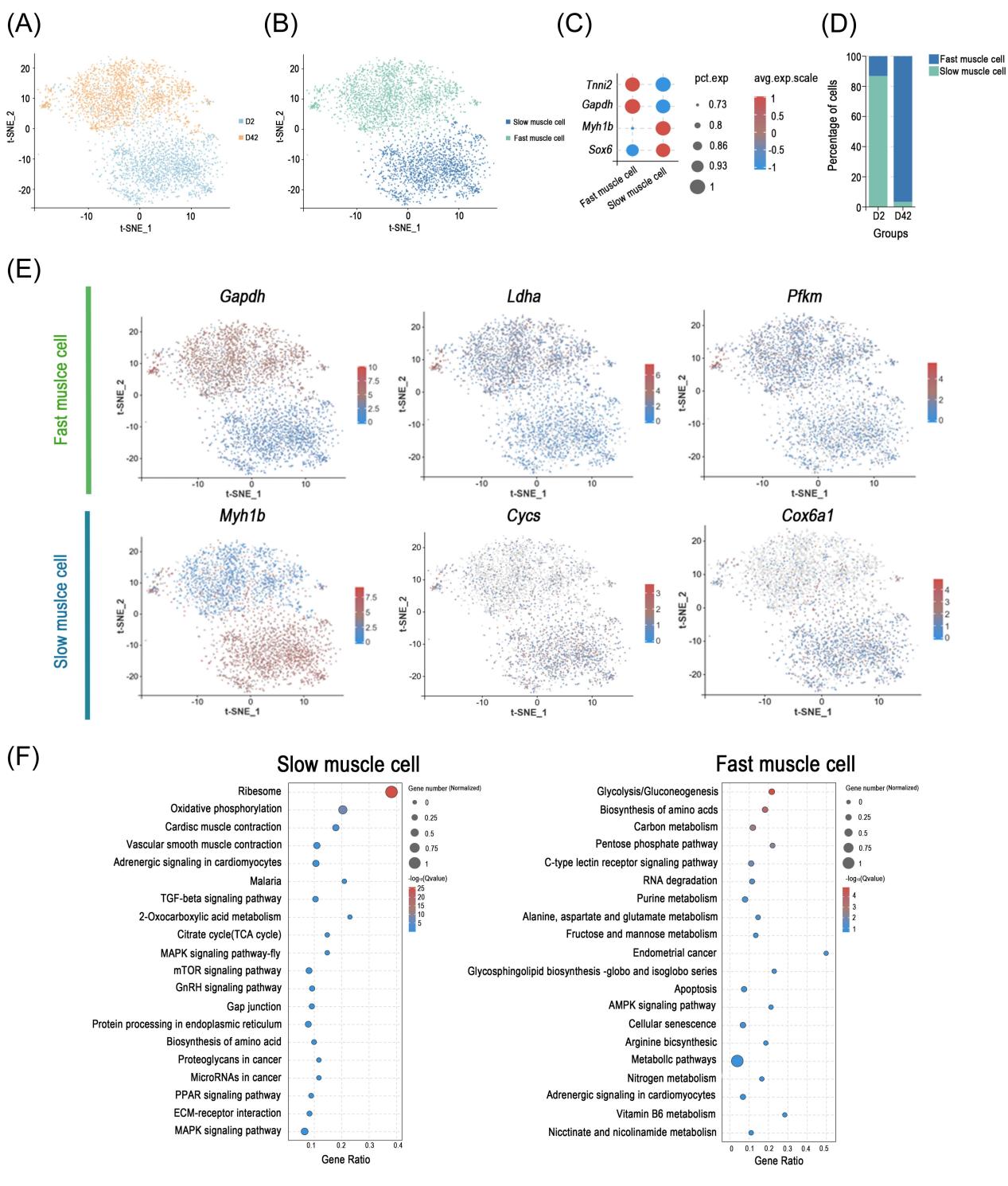


Figure S3. Characterization of the myocyte subpopulations. (A) t-SNE plots showing the distribution of the myocyte subpopulations by developmental stages (left) and (B) cell types (right). (C) Bubble plots of the mean expression of marker genes for myocyte subpopulations. (D) Bar plot showing the percentage of different myocyte subpopulations within each sample. (E) t-SNE plot showing the expression levels of representative genes in fast muscle cells and slow muscle cells. (F) KEGG analysis of the up-regulated differentially expressed genes in fast muscle cells and slow muscle cells.


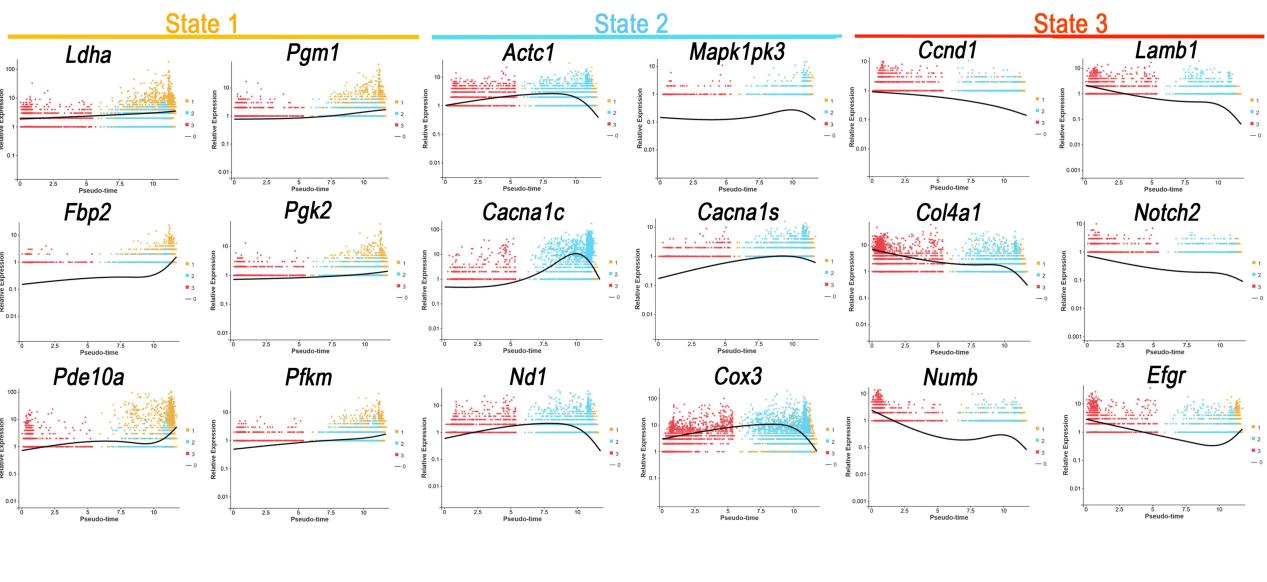


Figure S4. The dynamic expression of representative genes from differentiation states. Each dot represents a cell, different colors represent different states, the x-axis is the pseudotime value, and the y-axis is the gene expression level.

**
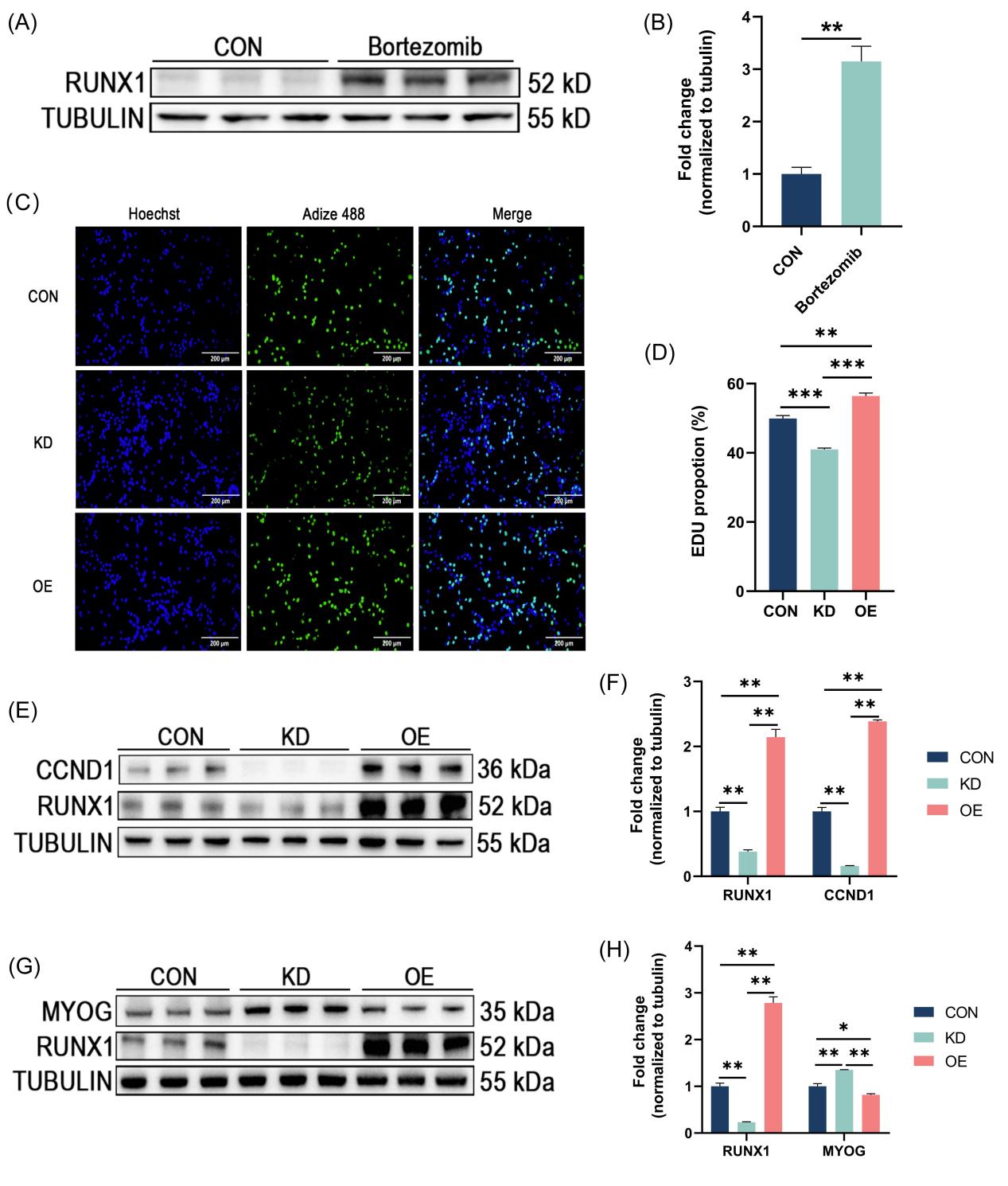
**

Figure S5. *Runx1* promotes myoblasts proliferation and inhibits differentiation. (A) Immunoblotting analysis and (B) quantification of total protein of RUNX1 in differentiated myoblasts treated by vehicle (DMSO) or Bortezomib. (C) EdU fluorescence staining (in green) and (D) percentage of EdU^+^ cells in CON (untreated) or *Runx1* KD (knockdown) and OE (overexpression) myoblasts, nuclei are counterstained with DAPI (in blue), Scale bars, 200 μm. (E) Immunoblotting analysis and (F) quantification of total protein of CCND1 in CON or *Runx1* KD and OE myoblasts at 0 dD (proliferation phase). (G) Immunoblotting analysis and (H) quantification of total protein of MYOG in CON or *Runx1* KD and OE myoblasts at day 1 of differentiation. Values are expressed as mean ± SEM (n = 3 or 6). The two-tailed Student's *t*-test and one-way ANOVA were used for two groups and three groups statistical analysis; After a significant ANOVA result, post hoc comparisons were performed using Duncan's multiple range test, and asterisks denote significant differences (**p* < 0.05, ***p* < 0.01 and ****p* < 0.001).


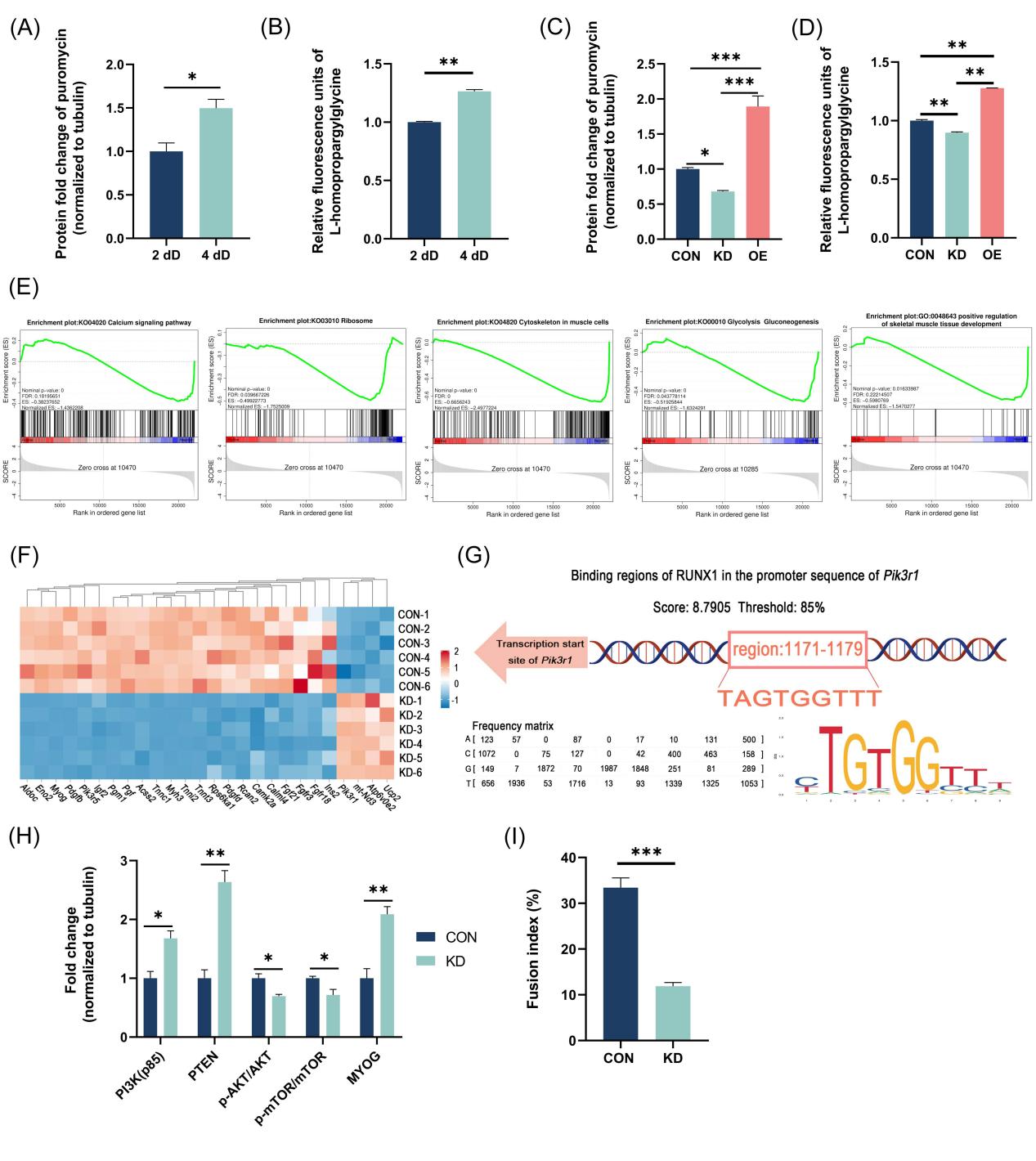


Figure S6. *Runx1* activates the PI3K/AKT/mTOR signaling pathway through transcriptional regulation of *Pik3r1*. (A) Quantification of total protein of puromycin in myoblasts at 2 dD (2 days of differentiation phase) and 4 dD (4 days of differentiation phase). (B) Normalized L-homopropargylglycine fluorescence intensity in myoblasts at 2 dD and 4 dD. (C) Quantification of total protein of puromycin in CON or *Runx1* KD and OE myoblasts at 4 dD. (D) Normalized L-homopropargylglycine fluorescence intensity in CON or *Runx1* KD and OE myoblasts at 4 dD. (E) Significantly enriched KEGG pathways identified by [GSEA](https://www.sciencedirect.com/topics/biochemistry-genetics-and-molecular-biology/gene-set-enrichment-analysis) (Gene Set Enrichment Analysis). (F) Heatmap displays the expression of representative differentially expressed genes in the CON and *Runx1* KD groups. |log2FC| ≥ 1 and *p* < 0.05 were set as the criteria. (G) Potential RUNX1-binding sites in the promoter region of *Pik3r1* gene using Jaspar database. The relative score threshold was set at 85%, and significantly enriched motifs are displayed as sequence logos. The frequency matrix represents the distribution frequency of the four nucleotides at each position within the motif. (H) Quantification of indicated proteins in CON and *Runx1* KD myoblasts at 4 dD. (I) Quantification of fusion index (a MYHC^+^ cell with at least three nucleus). Values are expressed as mean ± SEM (n = 3 or 6). The two-tailed Student's *t*-test and one-way ANOVA were used for two groups and three groups statistical analysis; After a significant ANOVA result, post hoc comparisons were performed using Duncan's multiple range test, and asterisks denote significant differences (**p* < 0.05, ***p* < 0.01 and ****p* < 0.001).
